# Supplementary material for: Ficus dubia Latex Extract Induces Cell Cycle Arrest and Apoptosis by Regulating the NF-κB Pathway in Inflammatory Human Colorectal Cancer Cell Lines
Source: Cancers (Basel). 2022 May 27;14(11):2665. doi: 10.3390/cancers14112665 (PMC9179257; doi:10.3390/cancers14112665)
Supplement: Supplementary file 1 [file cancers-14-02665-s001.zip › cancers-1670736-file S1.pdf]

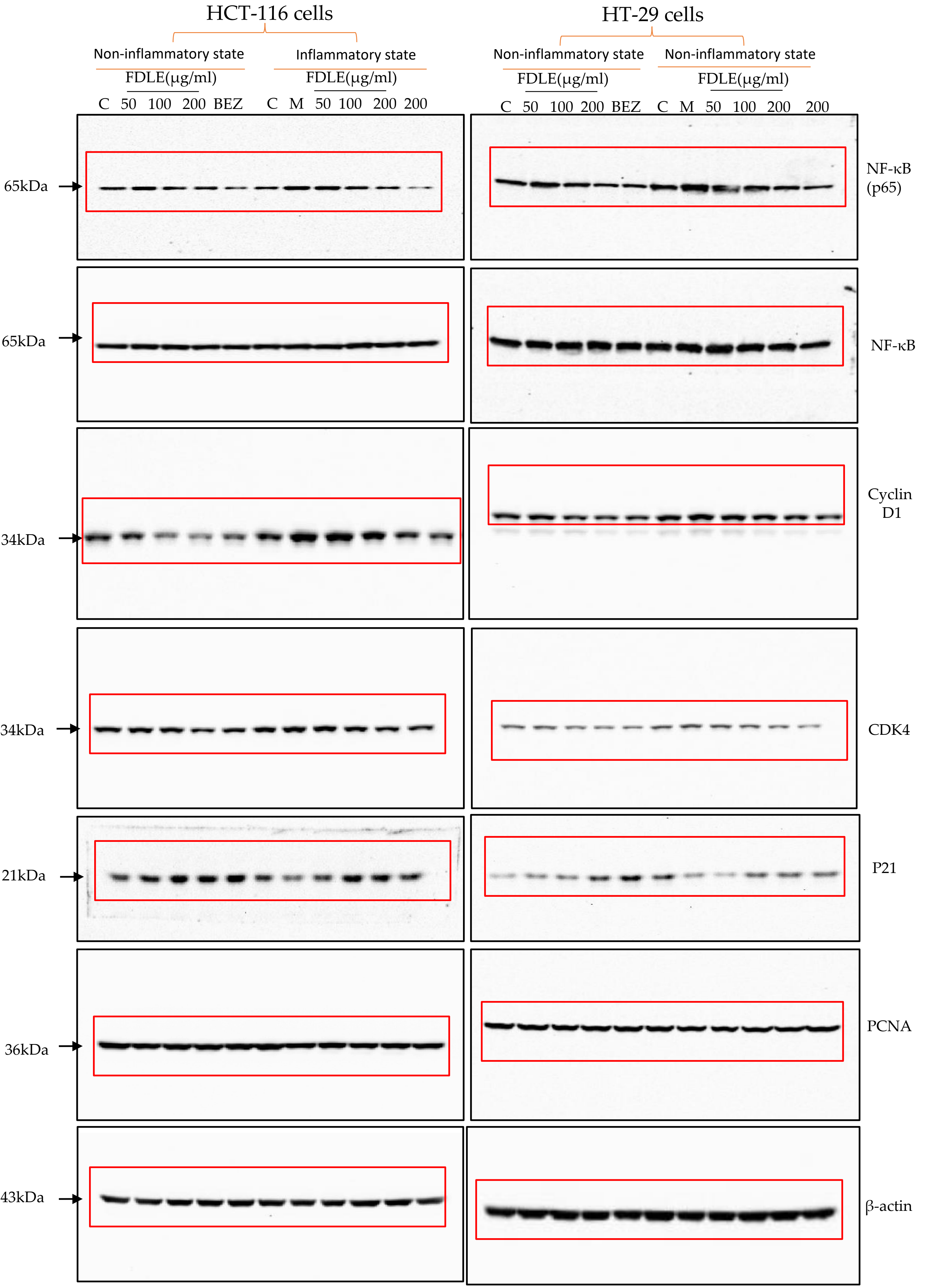

Supplementary Figure S1. Whole un-cropped images of the original western blots showing all bands with molecular weight markers. C: Negative control; BEZ: positive control; M: Mixture of cytokines. These gels correspond to those shown in Figure 5A-D.

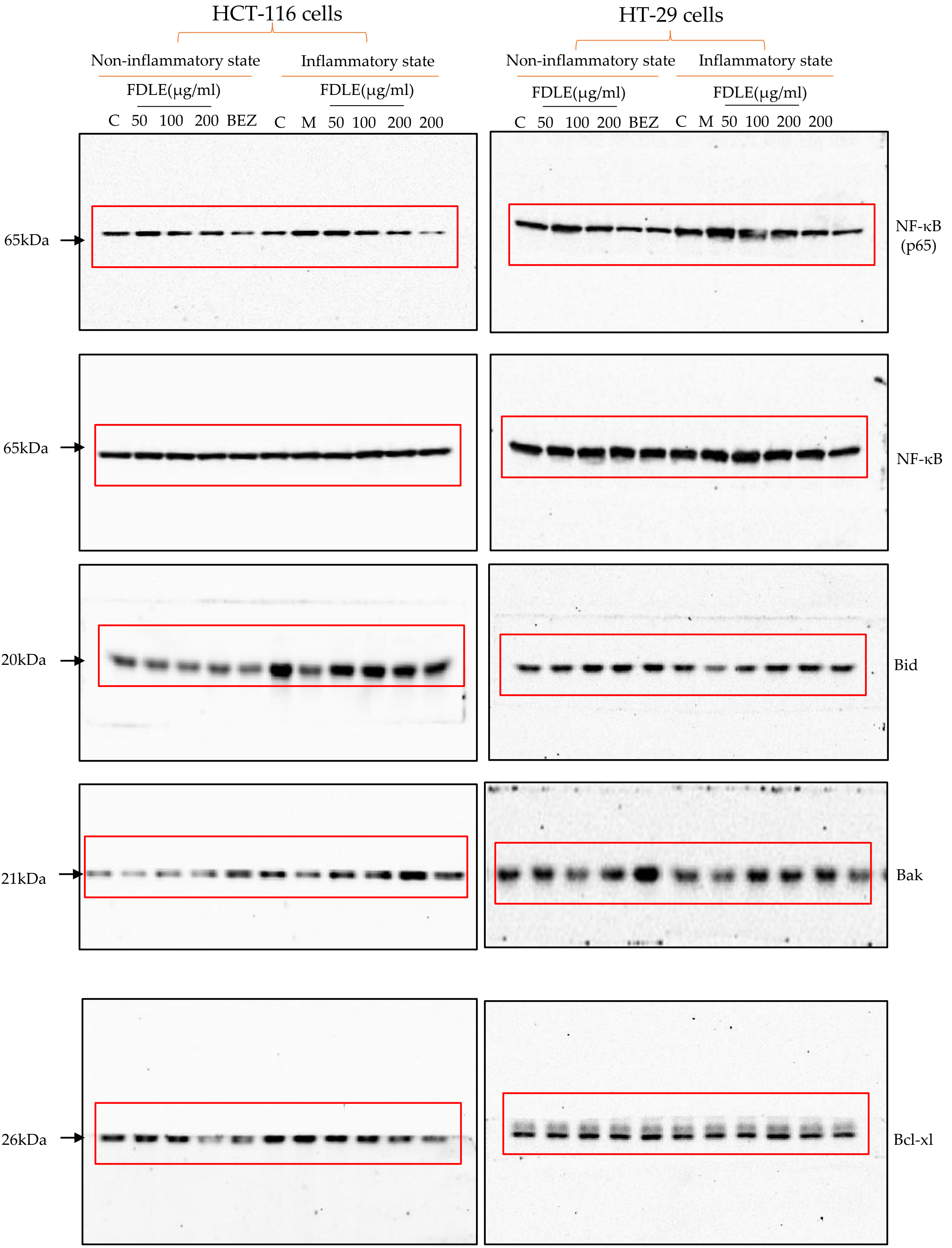

Supplementary Figure S2. Whole un-cropped images of the original western blots showing all bands with molecular weight markers. C: Negative control ; BEZ: positive control; M: Mixture of cytokines. These gels correspond to those shown in Figure 7A-D.

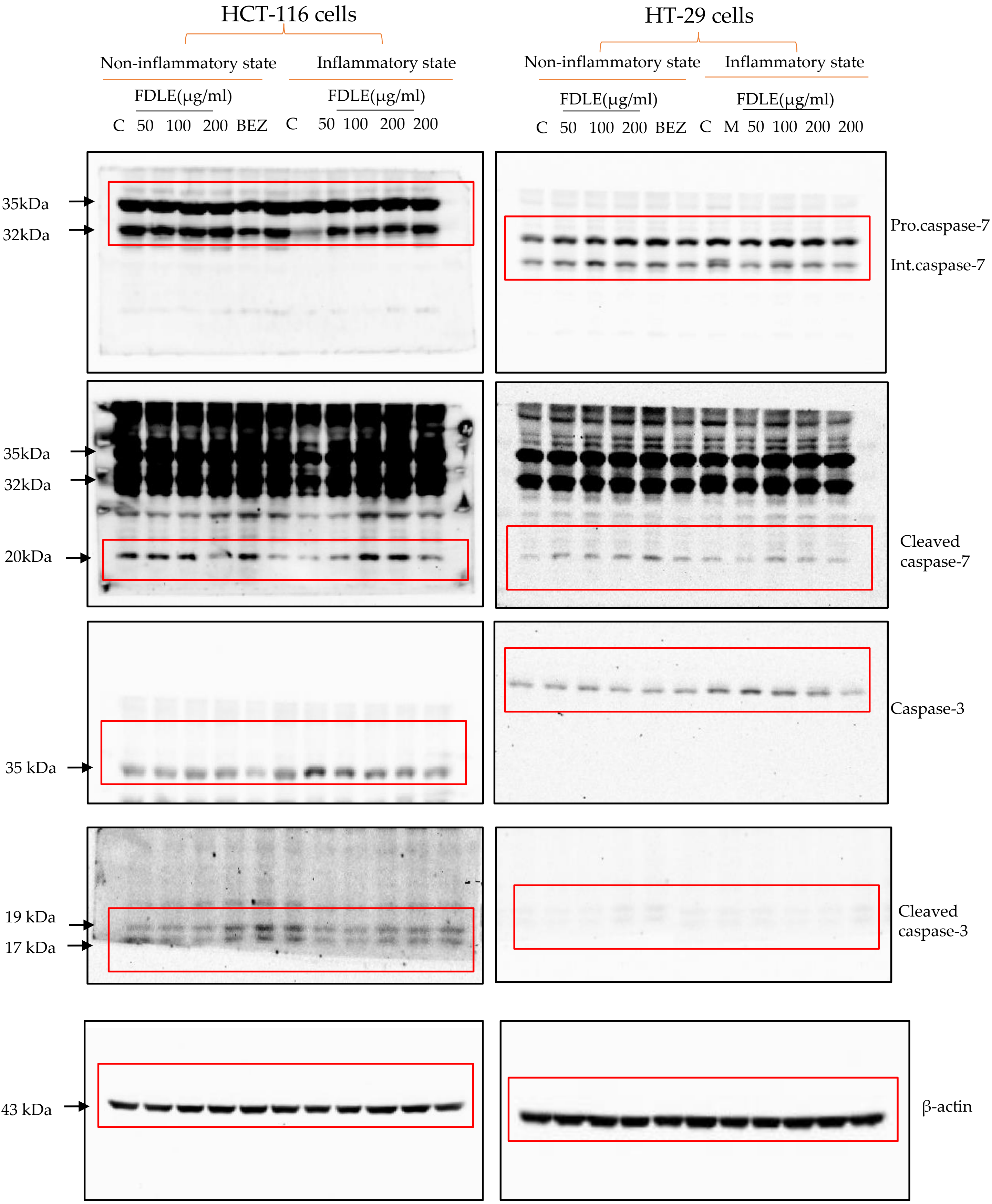

Supplementary Figure S3. Whole un-cropped images of the original western blots showing all bands with molecular weight markers. C: Negative control ; BEZ: positive control; M: Mixture of cytokines. These gels correspond to those shown in Figure 7A-D.
