# Supplementary material for: Ficus dubia Latex Extract Induces Cell Cycle Arrest and Apoptosis by Regulating the NF-κB Pathway in Inflammatory Human Colorectal Cancer Cell Lines
Source: Cancers (Basel). 2022 May 27;14(11):2665. doi: 10.3390/cancers14112665 (PMC9179257; doi:10.3390/cancers14112665)
Supplement: Supplementary file 1 [file cancers-14-02665-s001.zip › cancers-1670736-supplementary.pdf]

cancers

MDPI

Supplementary Materials

Ficus dubia latex extract induces cell cycle arrest and apoptosis by regulating the NF-κB pathway in inflammatory human colorectal cancer cell lines

Supplementary Table S1. The evaluation of IC20 and IC50 of FDLE on HCT-116 and HT-29 colorectal cancer cell lines and NIH3T3 normal cells in non-inflammatory state and inflammatory state.

	**HCT-116 in non-inflammatory state**		
	24 hours	48 hours	72 hours
IC_{20}	578.32±7.63	136.65±5.27	114.58±3.85
IC_{50}	>1000µg/mL	365.56±10.28	354.68±4.92
	**HCT-116 in inflammatory state**		
	24 hours	48 hours	72 hours
IC_{20}	512.86±6.12	50.35±5.74***	61.63±6.82**
IC_{50}	>1000µg/mL	105.81±7.35****	124.95±8.44***
	**HT-29 in non-inflammatory state**		
	24 hours	48 hours	72 hours
IC_{20}	423.15±8.36	130.43±5.68	116.17±7.42
IC_{50}	>1000µg/mL	330.72±11.26	370.55±9.81
	**HT-29 in inflammatory state**		
	24 hours	48 hours	72 hours
IC_{20}	553.64±7.22	64.94±4.12***	68.18±7.35**
IC_{50}	>1000µg/mL	104.13±6.54****	110.16±12.18****
	**NIH3T3 in non-inflammatory state**		
	24 hours	48 hours	72 hours
IC_{20}	>600µg/mL	>600µg/mL	>600µg/mL
IC_{50}	>1000µg/mL	>1000µg/mL	>1000µg/mL
	**NIH3T3 in inflammatory state**		
	24 hours	48 hours	72 hours
IC_{20}	>600µg/mL	136.85±8.26****	121.67±13.82****
IC_{50}	>1000µg/mL	246.61±12.65****	225.26±15.33****

* Data are presented as the Mean ± SD of three independent experiments which are similar results, **$p<0.01$, ***$p<0.001$and ****$p<0.0001$ vs. non-inflammatory cells in the same time.

Supplementary Table S2. The effect of the same concentration of FDLE on the inhibition ratio of colony formation for HCT-116 and HT-29 colorectal cancer cell lines in non-inflammatory state and inflammatory state.

FDLE(μg/mL)	Colony formation inhibition ratio(%)		Colony formation inhibition ratio(%)	
	Non-inflammatory HCT-116	Inflammatory HCT-116	Non-inflammatory HT-29	Inflammatory HT-29
50	16.63±1.22	19.28±1.85	17.36±2.12	21.39±3.54
100	39.81±1.48	39.40±6.13	39.50±5.43	36.34±6.12
200	47.79±3.28	58.45±2.23*	46.57±3.09	56.85±4.75*

* The inhibition ratio is relative to untreated group in the non-inflammatory state; The inhibition ratio is relative to cytokines-treated group in the inflammatory state, Data are presented as the Mean ± SD of three independent experiments which are similar results, *p<0.05 vs. non-inflammatory state in the same cells.

Supplementary Table S3. The effect of the same concentration of FDLE on the increased ratio of cell cycle arrest at GO/G1 phase in HCT-116 and HT-29 colorectal cancer cell lines in non-inflammatory state and inflammatory state.

FDLE(μg/mL)	Increased ratio of G0/G1(%)		Increased ratio of G0/G1(%)	
	Non-inflammatory HCT-116	Inflammatory HCT-116	Non-inflammatory HT-29	Inflammatory HT-29
50	0.39±2.00	11.4-±0.40***	1.40±1.61	7.80±1.56**
100	4.63±0.60	15.23±0.99****	3.30±2.01	12.10±1.87**
200	10.33±2.35	19.33±0.47**	9.13±2.07	16.60±2.01*

* The increased ratio of cell cycle arrest at GO/G1 phase were relative to untreated group in the non-inflammatory state; The increased ratio of cell cycle arrest at GO/G1 phase were relative to cytokines-treated group in the inflammatory state, Data are presented as the Mean ± SD of three independent experiments which are similar results, *p<0.05, **p<0.01, ***p<0.001and ****p<0.0001 vs. non-inflammatory state in the same cells.

Supplementary Table S4. To compare the relative change ratio of FDLE at high dose of 200μg/ml on proteins related to cell proliferation in non-inflammatory and inflammatory HCT-116 and HT-29 human colorectal cancer cell lines

Preoteins	Relative change ration in HCT-116 cells(%)		Relative change ration in HT-29 cells(%)	
	Non-inflammation (200μg/ml FDLE)	Inflammation (cytokines+200μg/ml FDLE)	Non-inflammation (200μg/ml FDLE)	Inflammation (cytokines+200μg/ml FDLE)
NF-κB(p65)	42.52±1.51 ↓	54.02±2.66** ↓	33.87±5.50 ↓	52.40±5.62* ↓
Cyclin D1	23.16±1.81 ↓	34.97±2.72** ↓	26.72±3.23 ↓	39.76±4.21* ↓
CDK4	17.90±4.90 ↓	23.34±4.50 ↓	41.04±3.84 ↓	54.89±4.00* ↓
P21	14.43±6.64 ↑	98.37±22.12** ↑	139.36±9.97 ↑	175.29±6.86* ↑
PCNA	4.69±9.09 ↓	6.74±16.40 ↓	0.01±8.20 ↓	8.99±5.73 ↓

The relative change ration in non-inflammation (200μg/ml FDLE) was compared to the untreated group. The relative change ration in inflammation (cytokines+200μg/ml FDLE) was compared to the cytokines-treated group. ↓: down-regulation, ↑: up-regulation. The results were represented as mean ± SD of three independent experiments, *p<0.05 and **p<0.01 vs. non-inflammation (200μg/ml FDLE) in the same cells.

Supplementary Table S5. The effect of the same concentration of FDLE on the increased ratio of apoptosis in HCT-116 and HT-29 colorectal cancer cell lines in non-inflammatory state and inflammatory state.

FDLE(μg/mL)	Increased ratio of apoptosis(%)		Increased ratio of apoptosis (%)	
	Non-inflammatory HCT-116	Inflammatory HCT-116	Non-inflammatory HT-29	Inflammatory HT-29
50	0.18±0.46	2.39±0.70*	0.11±0.16	0.06±0.09
100	0.11±0.02	6.32±1.03***	0.21±0.24	0.15±0.16
200	0.11±0.17	8.08±1.35***	0.05±0.34	0.03±0.12

* The increased ratio of apoptosis was relative to untreated group in the non-inflammatory state; The increased ratio of apoptosis was relative to cytokines-treated group in the inflammatory state, Data are presented as the Mean ± SD of three independent experiments which are similar results, *p<0.05 and ***p<0.05 vs. non-inflammatory state in the same cells.

Supplementary Table S6. To compare the relative change ratio of FDLE at high dose of 200μg/ml on proteins related to apoptosis in non-inflammatory and inflammatory HCT-116 and HT-29 human colorectal cancer cell lines

Preoteins	Relative change ration in HCT-116 cells(%)		Relative change ration in HT-29 cells(%)	
	Non-inflammation (200μg/ml FDLE)	Inflammation (cytokines+200μg/ml FDLE)	Non-inflammation (200μg/ml FDLE)	Inflammation (cytokines+200μg/ml FDLE)
Bid	13.64±11.34 ↑	70.08±6.10*** ↑	15.63±7.71 ↑	67.92±11.99** ↑
Bak	5.41±5.54 ↑	123.12±40.20** ↑	11.25±4.66 ↑	43.78±10.57* ↑
Bcl-xL	25.50±8.30 ↓	9.34±5.24 ↓	6.41±6.71 ↓	3.61±5.44 ↓
Clv. caspase-7	6.78±7.63 ↑	108.43±19.40** ↑	4.34±4.48 ↑	5.36±6.09 ↑
Int. caspase-7	0.24±6.20 ↑	67.17±7.46*** ↑	5.83±7.32 ↑	15.45±13.04 ↑
Clv. caspase-3	2.87 ±4.97 ↑	39.49±15.12** ↑	12.82±6.35 ↑	7.94±1.39 ↑

The relative change ration in non-inflammation (200μg/ml FDLE) was compared to the untreated group. The relative change ration in inflammation (cytokines+200μg/ml FDLE) was compared to the cytokines-treated group. ↓: down-regulation, ↑: up-regulation. The results were represented as mean ± SD of three independent experiments, *p<0.05, **p<0.01 and ***p<0.001 vs. non-inflammation (200μg/ml FDLE) in the same cells.

.
